# The length of the warm ischemic interval in lung donation after circulatory death does not impact post-transplantation outcomes

**DOI:** 10.1016/j.jhlto.2025.100244

**Published:** 2025-03-11

**Authors:** Amer Alzahrani, Kentaro Noda, Ernest G. Chan, John P. Ryan, Masashi Furukawa, Pablo G. Sanchez

**Affiliations:** a Division of Cardiothoracic Transplantation, Department of Cardiothoracic Surgery, University of Pittsburgh, Pittsburgh, PA; b Section of Thoracic Surgery, Department of Surgery. University of Chicago Biological Sciences Division, Chicago, IL; c Lung Health Centre Department, Organ Transplant Centre of Excellence, King Faisal Specialist Hospital and Research Centre, Riyadh, Saudi Arabia

**Keywords:** Lung Transplantation, Donation after Circulatory Death, Warm Ischemic Time, Risk Factors, Organ Donation, Donor Lungs

## Abstract

**Introduction:**

Transplantation of lungs obtained by donation after circulatory death (DCD) has increased the number of available organs. This study aims to determine how donor characteristics and current procurement processes (specifically, agonal and warm ischemic times) influence the outcomes experienced by the recipients of DCD lung transplants.

**Materials and Methods:**

An analysis was conducted on United Network for Organ Sharing data collected from January 2018 to June 30, 2024, with a focus on adult recipients of double lung transplants with a DCD donor. Withdrawal-to-flush and agonal-to-flush times were divided into three non-overlapping intervals. Univariable comparisons were performed on donor and recipient characteristics and post-transplantation outcomes between intervals. Kaplan-Meier analyses were used to determine the impact of agonal and warm ischemic times on posttransplant survival.

**Results:**

The median times for withdrawal-to-flush and agonal-to-flush were 28 and 25 minutes, respectively, with closely aligned intervals. Donors in the short agonal-to-flush category were generally older and tended to be female, with no other significant donor characteristics associated with the time intervals. There were no observed associations between agonal or warm ischemic times and post-transplant outcomes, including primary graft dysfunction, ventilator dependency, and acute rejection. Kaplan-Meier survival analysis revealed no significant differences in survival between the groups (p=0.47 for withdrawal-to-flush; p=0.57 for agonal-to-flush).

**Conclusions:**

This study suggests that current variations in withdrawal-to-flush and agonal-to-flush times are not associated with DCD lung transplant outcomes. The findings underscore the need for expanding strategies to increase the utilization and availability of DCD lungs.

## Introduction

Lung transplantation is a life-saving intervention for selected patients presenting with end-stage lung disease.^1^ Because of the significant mismatch between the number of lungs available from donation after brain death (DBD) donors and the pool of potential recipients,^2^, ^3^ Lungs obtained through donation after circulatory death (DCD) are an underutilized resource with the potential to significantly expand the donor pool and increase lung transplantation (LTx) numbers. Despite their similar favorable long-term survival, a large pool of DCD lungs remains untapped for transplantation.^4^, ^5^, ^6^

The increased adoption of DCD lung donations in the United States (US), alongside comparable outcomes in DBD transplants represent a significant paradigm shift in organ donation.^7^, ^8^ However, DCD lungs are used far less frequently for transplantation in the United States compared to DBD lungs, comprising only 4.2% of lung transplants. This gap stems from variations in donor evaluations, which make DCD donors less likely to provide lungs for transplantation than DBD donors.^9^, ^10^ In contrast, countries such as Australia, the United Kingdom, and the Netherlands have significantly higher utilization rates for donation after circulatory death (DCD) in lung transplantation, ranging from 30–50%. Within Europe, the practice of DCD is particularly notable, with Spain reporting a rate of 22.1 per million population (pmp), Belgium 18.0 pmp, the United Kingdom 11.1 pmp, and the Netherlands 11.0 pmp.^10^, ^11^, ^12^

One potential concern that centers may have with lungs originating from DCD donors is due to the extended warm ischemic time associated with DCD donation. In most countries, the threshold for suitability is set at less than 60 minutes, but in Australia, this limit has been extended to 90 minutes.^13^, ^14^, ^15^, ^16^, ^17^, ^18^, ^19^ In the US, practices among institutions vary due to state regulations, institutional policies, and the comfort level of the transplant team, which is increasingly comfortable with durations exceeding 60 minutes.^20^ Recently, initiatives have been observed to extend waiting durations to more than 90 minutes.^21^, ^22^

A detailed assessment of the International Society of Heart and Lung Transplant (ISHLT) DCD registry reports in 2015 and 2019 found no association between agonal or total warm ischemic times and one-year survival rates.^23^, ^24^ Both studies serve as foundational evaluations of warm ischemia times in DCD cases, yet they present certain limitations. A key challenge lies in the variability of definitions for different ischemia phases and acceptable warm ischemia durations across various regions,^25^ which reflects differing regional practices. Furthermore, the studies lack essential postoperative outcome data, such as the incidence of severe primary graft dysfunction (PGD3), treatments for acute rejection, and other outcomes beyond survival that crucially influence the quality of life following transplant.^23^, ^24^

The study aims to examine the relationship between agonal and warm ischemic times during lung procurement in DCD donors and their association with post-transplant outcomes in adult lung transplant recipients. By utilizing a national registry dataset from the United Network for Organ Sharing, we calculated intervals of withdrawal-to-flush duration and agonal-to-flush durations and examined associations with post-transplant outcomes. We hypothesized that prolonged warm ischemic times would not significantly impact post-transplant morbidity and mortality. By analyzing this national registry, we aim to assess whether extended warm ischemia plays an adverse role in DCD lung transplantation outcomes.

## Materials and methods

### Study design

This retrospective cohort study was based on data obtained from the United Network for Organ Sharing (UNOS) database based on the Organ Procurement and Transplantation Network (OPTN). Inclusion criteria were adults (≥ 18 years of age) with no history of prior transplant who underwent double lung transplantation between January 1, 2018, and June 30, 2024, and received lungs from a DCD donor. Cases involving multiorgan transplants were excluded. Donor data was linked to recipient data via donor identification codes. The study was approved by the University of Pittsburgh Human Research Protection Office (STUDY20050181).

### Data processing

Variables were used in the raw format provided by UNOS with some exceptions. Severe primary graft dysfunction (PGD3) was defined if the recipient had PaO2/FiO2 < 200 or was on ECMO at 72 hours post-transplant.^26^ Annual center volume was calculated by counting the number of lung transplant cases per center for each year. Lung discards were identified by limiting the dataset to donors who had two recovered for match but then at least one lung discarded.

**Figure 1:**
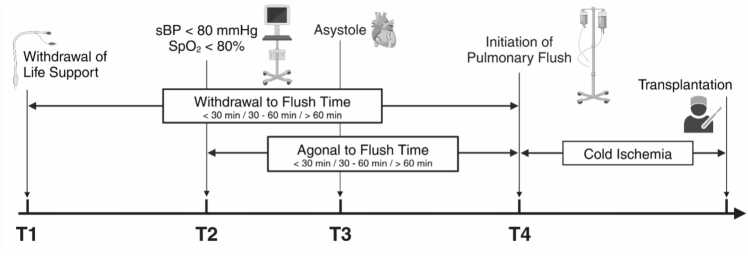
Diagram documenting the critical time points in the DCD lung procurement procedure and the time intervals evaluated in this study (withdrawal-to-flush and agonal-to-flush).

Procurement procedure times were determined by identifying the time of withdrawal of support, time of death, clamp time, and flush time in the deceased donor data file. We employed specific markers designated as T1 (the moment life support was withdrawn), T2 (the point at which systolic blood pressure declined to < 80 mmHg or oxygen saturation [SpO_2_] decreased to 80%), T3 (the time of asystole), and T4 (the commencement of pulmonary artery flush) as illustrated in Figure 1.

The investigation focuses on two principal time periods of warm ischemia during procurement:

1. Withdrawal-to-flush time (T1 to T4): The T1 to T4 intervals were divided into three non-overlapping durations: short (<30 minutes); medium (30 to 60 minutes); and long (> 60 minutes).
2. Agonal-to-flush time (T2 to T4): This interval captures the period from the initial clinical decline to the initiation of preservation measures (i.e., pulmonary artery flush) and was also divided into three non-overlapping durations: short (<30 minutes); medium (30 to 60 minutes); and long (> 60 minutes).

### Statistical analysis

Donor and recipient characteristics were compared across the three durations within each ischemic period using univariable comparisons. Comparisons were performed using Pearson’s chi-square and Fisher’s exact tests for categorical variables and Kruskal-Wallis rank sum tests. Post hoc comparisons of significant differences were performed by examining adjusted standardized residuals for categorical comparisons and by Dunn’s test for continuous variables. Kaplan-Meier analyses with log-rank tests were performed to investigate the effects of the group on posttransplant survival time. A supplementary Cox proportional hazard regression was conducted with agonal time as a continuous variable. Annual center volume was included as a nuisance covariate in the regression. To examine if higher agonal time was associated with greater probability of lung discard, a logistic regression was performed. Due to the amount of skew in agonal times, the times were log-transformed prior to the logistic regression to improve model fit. All analyses were performed in R (version 4.4.2); univariable comparisons were performed using the gtsummary package.^27^ A p-value of < 0.05 was considered statistically significant.

## Results

### Sample

Eight-hundred sixty-seven donors met the inclusion criteria. Of the 867, 550 (63.4%) had short agonal-to-flush durations, 169 (19.5%) had medium durations, and 148 (17.1%) had long durations. The median times from withdrawal-to-flush and agonal-to-flush were closely aligned, with overall median times of 28 minutes for withdrawal-to-flush (IQR: 23, 43) and 25 minutes for agonal-to-flush (IQR: 19, 37). There were significant differences across the three time periods for each metric. For withdrawal-to-flush, the median times were 23 minutes (IQR: 20, 26) for the short duration, 35 minutes (IQR: 31, 44) for the medium duration, and 92 minutes (IQR: 72, 128) for the long duration. Similarly, for agonal-to-flush, the median times were 21 minutes (IQR: 18, 24), 38 minutes (IQR: 32, 48), and 99 minutes (IQR: 74, 129) in the short, medium, and long durations, respectively (Table 1).

**Table 1:** Withdrawal to Flush and Agonal to Flush Times by Category

		Duration (Minutes)		
Characteristic	Overall	< 30	30-60	> 60
Withdrawal to Flush Time (minutes)				
Median (Q1, Q3)	28 (23, 43)	23 (20, 26)	35 (31, 44)	92 (72, 128)
N Non-missing	557	311	145	101
Agonal to Flush Time (minutes)				
Median (Q1, Q3)	25 (19, 37)	21 (18, 24)	38 (32, 48)	99 (74, 129)
N Non-missing	559	371	101	87

### Characteristics of DCD lung donors

There were several differences in donor characteristics across the three agonal-to-flush durations (Table 2). Donors with short durations were older (median 43 years) compared to those in the medium (median 40 years) and long (median 36 years; p < 0.001) duration groups. Donor sex was also significantly associated with agonal-to-flush times, with female donors more likely to be in the medium duration group (47%) but less likely to be in the long duration group (32%; p = 0.03). The medium duration group also had a higher-than-expected proportion of donors > 55 years old (23%) relative to the short and long duration groups (13% and 3.4%, respectively, p < 0.001). There was no association between agonal-to-flush duration and creatinine level, pulmonary infection rate, history of cigarette or heavy alcohol use, diabetes, or other donor characteristics.

**Table 2:** Donor Characteristics of DCD Donors

	Agonal to Flush Time (mins)			
Characteristic	< 30 N = 550	30-60 N = 169	> 60 N = 148	p-value^a^
Donor Age (years), Median (IQR)	40 (28 – 50)^a^	43 (33 – 52)^b^	36 (26 – 47)^c^	<0.001
Donor Sex, n (%)				0.030
Female	232 (42)	79 (47)	48 (32)*	
Male	318 (58)	90 (53)	100 (68)*	
Creatinine (mg/dL), Median (IQR)	0.78 (0.60 – 1.07)	0.74 (0.60 – 1.00)	0.74 (0.60 – 0.98)	0.67
Pulmonary Infection, n (%)	359 (65)	108 (64)	103 (70)	0.53
Cigarette History, n (%)	57 (11)	19 (11)	8 (5.5)	0.15
Heavy Alcohol History, n (%)	131 (25)	46 (28)	44 (31)	0.26
Diabetes, n (%)	42 (11)	11 (11)	4 (4.7)	0.17
Purulent Bronchoscopy, n (%)	59 (16)	18 (18)	12 (14)	0.75
Abnormal X-Ray, n (%)	238 (64)	67 (67)	61 (70)	0.59
PO2 < 300, n (%)	72 (19)	19 (19)	15 (17)	0.90
Age > 55 years, n (%)	50 (13)	23 (23)	3 (3.4)*	<0.001
Extended Criteria, n (%)	67 (12)	26 (15)	11 (7.4)	0.092
Cause of Brain Injury, n (%)				0.36
Anoxia	234 (44)	61 (37)	68 (47)	
CNS Tumor	2 (0.4)	0 (0)	0 (0)	
CVA	141 (26)	50 (31)	29 (20)	
Head Trauma	159 (30)	52 (32)	47 (33)	
Distance Donor Hosp to TX Center (Nautical Miles), Median (IQR)	231 (99 – 552)	204 (65 – 486)	176 (25 – 498)	0.10

a Kruskal-Wallis rank sum test; Pearson's Chi-squared test; Fisher's exact test

### Characteristics of DCD lung recipients

There were no significant differences in recipient characteristics across the three agonal-to-flush duration categories (Table 3). Recipients were similar in age, sex and indication for transplant, had similar health histories (alcohol and smoking), similar serum creatinine, as well as pre-transplant bridge support and lung allocation score at the time of transplant. There was not a significant association between the agonal-to-flush duration category and the time the recipient had been on the waitlist. Notably, there was not a significant difference between the three agonal-to-flush duration categories in total ischemic time with each group having relatively similar total ischemic time (7.7–7.9 hours, p = 0.46).

**Table 3:** Recipient Characteristics

	Agonal to Flush Time (mins)			
Characteristic	< 30 N = 371	30-60 N = 101	> 60 N = 87	p-value^a^
Recipient Age, Median (IQR)	62 (54 – 67)	61 (56 – 66)	62 (56 – 65)	0.90
Sex, n (%)				0.38
Female	166 (45)	53 (52)	40 (46)	
Male	205 (55)	48 (48)	47 (54)	
Diagnosis, n (%)				0.83
Obstructive	108 (29)	32 (32)	30 (34)	
Pulmonary Hypertension	12 (3.2)	5 (5.0)	3 (3.4)	
Restrictive	235 (63)	62 (61)	51 (59)	
Suppurative	16 (4.3)	2 (2.0)	3 (3.4)	
Body Mass Index (kg/m2), Median (IQR)	26.3 (22.7 – 29.2)	26.5 (22.2 – 29.8)	25.6 (22.7 – 29.0)	0.84
Cigarette History, n (%)	38 (10)	11 (11)	3 (3.5)	0.12
Diabetes, n (%)	42 (11)	11 (11)	4 (4.7)	0.17
Steroids, n (%)	62 (19)	17 (22)	22 (30)	0.15
O2 Requirement at Rest (L/min), Median (IQR)	4 (2 – 6)	4 (2 – 6)	4 (2 – 6)	0.91
Serum Creatinine (mg/dL), Median (IQR)	0.80 (0.65 – 0.94)	0.82 (0.69 – 0.94)	0.76 (0.64 – 0.95)	0.68
Condition at Transplant, n (%)				0.39
Hospitalized, Non-ICU	31 (8.7)	10 (9.9)	10 (13)	
ICU	46 (13)	7 (6.9)	11 (14)	
Not Hospitalized	279 (78)	84 (83)	59 (74)	
Ventilator Bridge to Transplant, n (%)	13 (3.5)	1 (1.0)	5 (5.7)	0.20
ECMO Bridge to Transplant, n (%)	18 (4.9)	3 (3.0)	4 (4.6)	0.82
Lung Allocation Score at Transplant, Median (IQR)	41 (35 – 53)	39 (35 – 46)	39 (34 – 47)	0.53
Time on Waitlist (days), Median (IQR)	34 (11 – 104)	30 (10 – 97)	34 (12 – 85)	0.98
Ischemic Time (hours), Median (IQR)	7.7 (6.1 – 10.3)	7.9 (6.0 – 12.6)	7.7 (5.2 – 12.5)	0.45
EVLP Lungs, n (%)	81 (23)	30 (30)	23 (29)	0.26
Annual Center Volume, Median (IQR)	64 (41 – 94)	60 (47 – 90)	54 (37 – 81)	0.21

a Kruskal-Wallis rank sum test; Pearson's Chi-squared test; Fisher's exact test

### Post transplantation outcomes

An examination of post-transplant outcomes across the three agonal-to-flush time groups revealed no significant associations between flush durations and key outcomes (Table 4). Rates of PGD3 were similar across the durations, with 23% in the short duration group, 21% in the medium duration group, and 15% in the long duration group (p = 0.24). Most patients required ventilator support for 48 hours or less, and extended support durations did not vary significantly between the groups (p = 0.69). Airway dehiscence and stroke rates were low across all groups, with no statistically significant differences (p = 0.26 for airway dehiscence and p > 0.99 for stroke). The median length of stay was slightly longer in the medium duration group (27 days) compared to the short and long duration groups (22 and 20 days, respectively), but this difference did not reach statistical significance (p = 0.11). Similarly, rates of acute rejection within one year were low and consistent across the groups (<3%, p = 0.81).

**Table 4:** Post-Transplant Outcomes

		Agonal to Flush Time (mins)			
Characteristic	N	< 30 N = 371	30-60 N = 101	> 60 N = 87	p-value^a^
PGD3, n (%)	559	86 (23)	21 (21)	13 (15)	0.24
Ventilator Support, n (%)	531				0.69
None		6 (1.7)	3 (3.0)	1 (1.3)	
Ventilator support for <= 48 h		146 (42)	40 (40)	40 (51)	
Ventilator support for >48 h but < 5 days		82 (23)	23 (23)	13 (16)	
Ventilator support >= 5 days		117 (33)	35 (35)	25 (32)	
Airway Dehiscence, n (%)	535	2 (0.6)	2 (2.0)	0 (0)	0.26
Stroke, n (%)	535	10 (2.8)	2 (2.0)	2 (2.5)	>0.99
Dialysis, n (%)	536	38 (11)	17 (17)	7 (8.9)	0.17
Length of Stay, Median (IQR)	523	22 (16 – 41)	27 (18 – 50)	20 (14 – 38)	0.11
Treated for Acute Rejection Within 1 Year, n (%)	491	3 (0.9)	1 (1.1)	1 (1.4)	0.81

a Pearson's Chi-squared test; Fisher's exact test; Kruskal-Wallis rank sum test

### Post-transplant survival

**Figure 2:**
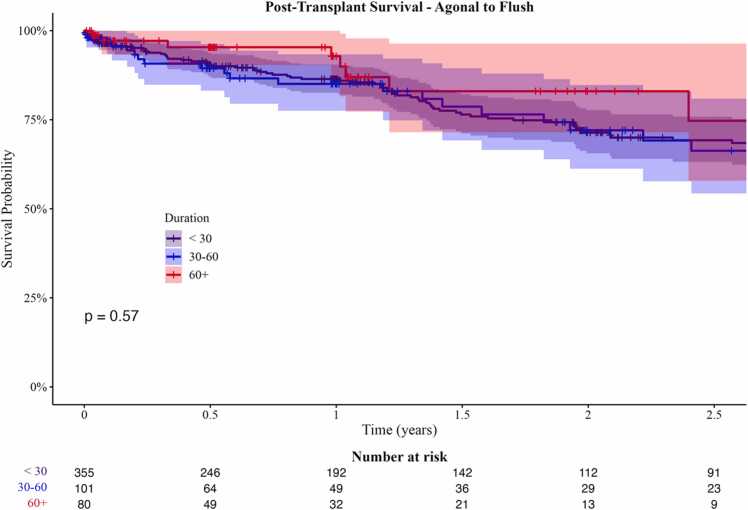
Kaplan-Meier survival curves for 54 months post-transplantation for each agonal-to-flush time interval evaluated.

The Kaplan-Meier method found no association between agonal-to-flush time and post-transplant survival time (χ^2^(2) = 1.12, p = 0.57, Figure 2). When times were kept as a continuous variable, there was still no relationship with post-transplant survival time (Supplemental Table S4).

### Withdrawal to flush times

Given the similarity between the findings for agonal-to-flush time and withdrawal-to-flush durations, the withdrawal-to-flush data are provided in the supplemental material.

### EVLP subgroup analysis

In the subgroup analysis of lungs that underwent perfusion prior to transplant, there were no significant differences between the three agonal-to-flush durations in rates of PGD3 (p = 0.63), post-transplant ventilator duration (p=0.58), airway dehiscence (p=0.26), or stroke (Table 5, p = 0.48). There was a significant association between agonal-to-flush duration and rates of post-transplant dialysis with recipients of EVLP lungs who had a short agonal-to-flush duration having lower than expected rates of post-transplant dialysis (6.2% vs. 17% and 23% in medium and long durations, respectively; p = 0.04).

**Table 5:** Post-Transplant Outcomes for EVLP Cases

		Agonal to Flush Time (mins)			
Characteristic	N	< 30 N = 81	30-60 N = 30	> 60 N = 23	p-value^a^
PGD3, n (%)	134	18 (22)	7 (23)	3 (13)	0.63
Ventilator Support, n (%)	130				0.58
None		1 (1.3)	2 (6.7)	1 (4.5)	
Ventilator support for <= 48 h		29 (37)	11 (37)	11 (50)	
Ventilator support for >48 h but < 5 days		20 (26)	7 (23)	3 (14)	
Ventilator support >= 5 days		28 (36)	10 (33)	7 (32)	
Airway Dehiscence, n (%)	133				
No		81 (100)	30 (100)	22 (100)	
Stroke, n (%)	133	4 (5.0)	0 (0)	0 (0)	0.48
Dialysis, n (%)	133	5 (6.2)*	5 (17)	5 (23)	0.042
Length of Stay, Median (IQR)	132	22 (16 – 48)	31 (18 – 53)	35 (22 – 50)	0.32
Treated for Acute Rejection Within 1 Year, n (%)	122				
No		74 (100)	28 (100)	20 (100)	

* p < 0.05 post hoc

a Fisher's exact test; Kruskal-Wallis rank sum test

### Agonal time and lung discards

To examine the relationship between agonal time and the probability of a lung being discarded after being recovered, discard status was regressed on agonal time. There was not a significant relationship between agonal-to-flush time and discard probability (odds ratio 1.20 (95% confidence interval 0.95 – 1.50), p = 0.12). There was a trend towards significance with withdrawal-to-flush times (odds ratio 1.25 (95% confidence interval 0.99–1.61), p = 0.056). If extended criteria variables were entered in as nuisance covariates, the results did not significantly change (data not shown).

## Discussion

Lungs from DCD donors remain an underutilized resource for transplant candidates, largely due to extended warm ischemic times, which may make transplant teams hesitant to consider DCD lungs. Decisions to proceed with lungs that have exceeded sixty minutes of warm ischemic time are influenced by factors such as the transplant team’s experience, confidence, and adherence to OPN guidelines. These variations introduce challenges in forming definitive conclusions regarding this practice. Historically, viability concerns for DCD lungs stemmed from experiences with organs like the liver and kidneys, where preservation within 30 minutes after life support cessation was deemed critical. 18, 22, 28, 29 However, clinical practices have since extended this threshold to as much as 120 minutes, based on the hypothesis that lungs have lower metabolic demands and oxygen storage capacity, which allows for longer warm ischemic periods.^30^

The present study suggests that current extended time intervals were not associated with increased mortality or adverse post-operative outcomes following lung transplantation.

While the acceptable withdrawal-to-flush interval has been defined by international consensus, the start of agonal time remains variable. In some trials, agonal time begins when systolic blood pressure falls below 50 mmHg, which contributes to inconsistencies in reporting these intervals.^23^, ^24^ The present study adhered to the UNOS definition of agonal time: systolic blood pressure declined to < 80 mmHg or oxygen saturation decreased to less than 80%. By aligning our criteria with these standards, we have obtained a clearer understanding of the impact of agonal-to-flush times on DCD lung transplant outcomes.

Our analysis demonstrated that median times, whether measured from withdrawal-to-flush or agonal-to-flush, were aligned across various duration categories, showing no major differences between the two ways of defining the intervals. Additionally, our assessment of post-transplant outcomes indicated that the timing of organ procurement—regardless of whether measured from withdrawal-to-flush or agonal-to-flush—had no significant effect on immediate or short-term complications following DCD transplantation. The lack of significant associations between these time intervals and post-operative outcomes suggests that the impact of extended warm ischemic times may have minimal impact on post-transplant outcomes, both in the immediate post-operative period as well as longer-term survival. This result is particularly important, as previous studies have not thoroughly documented key outcomes such as rates of PGD3, ventilator support, length of hospital length of stay, or other adverse outcomes including dialysis and acute rejection.^23^, ^24^ Moreover, our survival findings are consistent with previous research, including reports from the ISHLT database, which similarly found no significant association between flush durations and survival outcomes.^23^, ^24^

EVLP provides a unique opportunity to further evaluate DCD lungs that may otherwise be unsuitable for transplant. In a subgroup analysis of EVLP cases within our cohort, associations between warm ischemic time durations and post-transplant outcomes were largely similar. However, shorter agonal-to-flush times were protective against post-transplant dialysis. This finding could suggest that transplant candidates who are at higher risk for renal complications should carefully evaluate DCD lungs that have undergone EVLP – and if the agonal duration was short, the lungs could potentially confer a protective effect. Further studies will be needed to replicate this finding and explore potential mechanistic explanations.

The log-transformed analysis of agonal times showed a potential trend toward increased discard risk with longer intervals (p = 0.15 for agonal-to-flush, p = 0.056 for withdrawal-to-flush), though results were not statistically significant. Adding extended criteria covariates did not change the findings. These trends suggest that longer agonal times *may* slightly influence discard risk, warranting further investigation in larger datasets.

Several limitations of the study should be noted. First, the relatively small sample size may have limited the statistical power to detect significant differences in survival. Future studies with longer follow-up times will be needed to increase the power to detect any potential effects on survival. The observational nature of the study also restricts the ability to infer causality. Finally, there is variability in how DCD procurements are managed in different OPOs that may have created variability that was unmeasured in the present study. Differences in center-specific protocols and OPO management may influence outcomes, underscoring the need for future research to assess the impact of these factors on a national scale.

Extended warm ischemic time has been cited as a reason that centers may hesitate to utilize lungs from DCD donors. The study found no association between the length of withdrawal-to-flush or agonal-to-flush durations and post-transplant outcomes in lung transplant recipients. These findings suggest that lungs from DCD donors should continue to be considered by transplant teams as they are an underutilized resource for an organ that is in short supply.

## CRediT authorship contribution statement

AA, KN, and PGS designed the study. JR analyzed the data. AA prepared the initial draft of the manuscript. KN, EGC, JR, MF, and PGS contributed to the manuscript revisions. AA, KN, EGC, JR, MF, and PGS all provided final approval of the version of the manuscript to be submitted for publication.

## Declaration of Competing Interest

The authors declare that they have no competing financial interests or personal relationships that could influence the work reported in this paper. This research did not receive any specific grant from funding agencies in the public, commercial, or not-for-profit sectors.

## Footnotes

Appendix A Supplemental data associated with this article can be found in the online version at doi:10.1016/j.jhlto.2025.100244.
