# Supplementary material for: The length of the warm ischemic interval in lung donation after circulatory death does not impact post-transplantation outcomes
Source: JHLT Open. 2025 Mar 11;8:100244. doi: 10.1016/j.jhlto.2025.100244 (PMC12142563; doi:10.1016/j.jhlto.2025.100244)
Supplement: Supplementary file 1 — Supplemental material [file mmc1.docx]

**Supplementary Data:**

Table 1. Donor Characteristics of DCD Donors.

|  | Withdrawal-to-flush Time (mins) | | |  |
| --- | --- | --- | --- | --- |
| **Characteristic** | **< 30**  N = 451 | **30-60**  N = 243 | **>61**  N = 170 | **p-value**^1^ |
| Donor Age (years), Median (IQR) | 40 (28 – 51) | 41 (31 – 50) | 37 (26 – 48) | 0.074 |
| Donor Sex, n (%) |  |  |  | 0.26 |
| Female | 193 (43) | 104 (43) | 61 (36) |  |
| Male | 258 (57) | 139 (57) | 109 (64) |  |
| Creatinine (mg/dL), Median (IQR) | 0.80 (0.60 – 1.10) | 0.74 (0.60 – 1.00) | 0.74 (0.60 – 1.00) | 0.39 |
| Pulmonary Infection, n (%) | 291 (65) | 160 (66) | 117 (69) | 0.60 |
| Cigarette History, n (%) | 50 (11) | 25 (10) | 9 (5.4) | 0.087 |
| Heavy Alcohol History, n (%) | 109 (25) | 59 (25) | 53 (32) | 0.16 |
| Diabetes, n (%) | 33 (11) | 18 (13) | 6 (6.0) | 0.23 |
| Purulent Bronchoscopy, n (%) | 50 (16) | 26 (18) | 13 (13) | 0.57 |
| Abnormal X-Ray, n (%) | 205 (66) | 88 (61) | 72 (71) | 0.25 |
| PO2 < 300, n (%) | 61 (20) | 28 (19) | 17 (17) | 0.82 |
| Age > 55 years, n (%) | 43 (14) | 23 (16) | 10 (9.9) | 0.40 |
| Extended Criteria, n (%) | 53 (12) | 34 (14) | 17 (10) | 0.45 |
| Cause of Death, n (%) |  |  |  | 0.41 |
| Anoxia | 191 (43) | 93 (40) | 79 (48) |  |
| CNS Tumor | 2 (0.5) | 0 (0) | 0 (0) |  |
| CVA | 111 (25) | 72 (31) | 36 (22) |  |
| Head Trauma | 136 (31) | 69 (29) | 51 (31) |  |
| Distance Donor Hosp to TX Center (Nautical Miles), Median (IQR) | 228 (98 – 547) | 213 (88 – 536) | 214 (29 – 498) | 0.46 |
| ^1^Kruskal-Wallis rank sum test; Pearson's Chi-squared test; Fisher's exact test | | | | |

Table 2. Recipient Characteristics.

|  | Withdrawal-to-flush Time (mins) | | |  |
| --- | --- | --- | --- | --- |
| **Characteristic** | **< 30**  N = 311 | **30-60**  N = 145 | **>61**  N = 101 | **p-value**^1^ |
| Recipient Age, Median (IQR) | 61 (54 – 66) | 63 (56 – 67) | 62 (55 – 65) | 0.15 |
| Sex, n (%) |  |  |  | 0.89 |
| Female | 146 (47) | 65 (45) | 48 (48) |  |
| Male | 165 (53) | 80 (55) | 53 (52) |  |
| Diagnosis, n (%) |  |  |  | 0.97 |
| Obstructive | 95 (31) | 44 (30) | 31 (31) |  |
| Pulmonary Hypertension | 10 (3.2) | 7 (4.8) | 3 (3.0) |  |
| Restrictive | 193 (62) | 90 (62) | 63 (62) |  |
| Suppurative | 13 (4.2) | 4 (2.8) | 4 (4.0) |  |
| Body Mass Index (kg/m2), Median (IQR) | 26.1 (22.4 – 29.0) | 26.7 (23.0 – 29.8) | 25.8 (22.7 – 29.0) | 0.49 |
| Diabetes, n (%) | 33 (11) | 18 (13) | 6 (6.0) | 0.23 |
| Steroids, n (%) | 53 (20) | 22 (19) | 26 (31) | 0.066 |
| O2 Requirement at Rest (L/min), Median (IQR) | 4.0 (2.0 – 6.0) | 4.0 (2.0 – 6.0) | 4.0 (2.0 – 6.0) | 0.68 |
| Serum Creatinine (mg/dL), Median (IQR) | 0.78 (0.63 – 0.91)^a^ | 0.83 (0.70 – 0.99)^b^ | 0.75 (0.66 – 0.95)^a,b^ | 0.046 |
| Condition at Transplant, n (%) |  |  |  | 0.10 |
| Hospitalized, Non-ICU | 24 (7.9) | 12 (8.6) | 14 (15) |  |
| ICU | 42 (14) | 11 (7.9) | 11 (12) |  |
| Not Hospitalized | 236 (78) | 117 (84) | 68 (73) |  |
| Ventilator Bridge to Transplant, n (%) | 10 (3.2) | 4 (2.8) | 5 (5.0) | 0.63 |
| ECMO Bridge to Transplant, n (%) | 15 (4.8) | 6 (4.1) | 4 (4.0) | 0.96 |
| Lung Allocation Score at Transplant, Median (IQR) | 41 (36 – 57) | 38 (34 – 45) | 39 (34 – 47) | 0.064 |
| Time on Waitlist (days), Median (IQR) | 37 (12 – 106) | 27 (8 – 97) | 37 (12 – 87) | 0.31 |
| Ischemic Time (hours), Median (IQR) | 7.6 (6.0 – 10.2) | 8.2 (6.1 – 11.9) | 7.8 (5.4 – 11.4) | 0.36 |
| EVLP Lungs, n (%) | 68 (23) | 39 (28) | 27 (29) | 0.31 |
| ^1^Kruskal-Wallis rank sum test; Pearson's Chi-squared test; Fisher's exact test  a,b – Columns with different superscripts are significantly different post hoc | | | | |

Table 3. Post-Transplant Outcomes.

|  | | Withdrawal-to-flush Time (mins) | | |  |
| --- | --- | --- | --- | --- | --- |
| **Characteristic** | **N** | **< 30**  N = 311 | **30-60**  N = 145 | **>61**  N = 101 | **p-value**^1^ |
| PGD3, n (%) | 557 | 74 (24) | 31 (21) | 15 (15) | 0.16 |
| Ventilator Support, n (%) | 529 |  |  |  | 0.43 |
| Ventilator support for <= 48 hours |  | 120 (40) | 58 (42) | 48 (52) |  |
| Ventilator support for >48 hours but < 5 days |  | 73 (24) | 30 (22) | 14 (15) |  |
| Ventilator support >= 5 days |  | 100 (34) | 48 (35) | 28 (30) |  |
| Airway Dehiscence, n (%) | 533 | 2 (0.7) | 2 (1.4) | 0 (0) | 0.64 |
| Stroke, n (%) | 533 | 9 (3.0) | 3 (2.1) | 2 (2.2) | 0.93 |
| Dialysis, n (%) | 534 | 35 (12) | 18 (13) | 9 (9.8) | 0.77 |
| Length of Stay, Median (IQR) | 521 | 23 (16 – 43) | 25 (16 – 52) | 20 (14 – 38) | 0.15 |
| Treated for Acute Rejection Within 1 Year, n (%) | 489 | 3 (1.1) | 1 (0.8) | 1 (1.2) | >0.99 |
| ^1^Pearson's Chi-squared test; Fisher's exact test; Kruskal-Wallis rank sum test |  |  |  |  |  |
|  | | | | | |


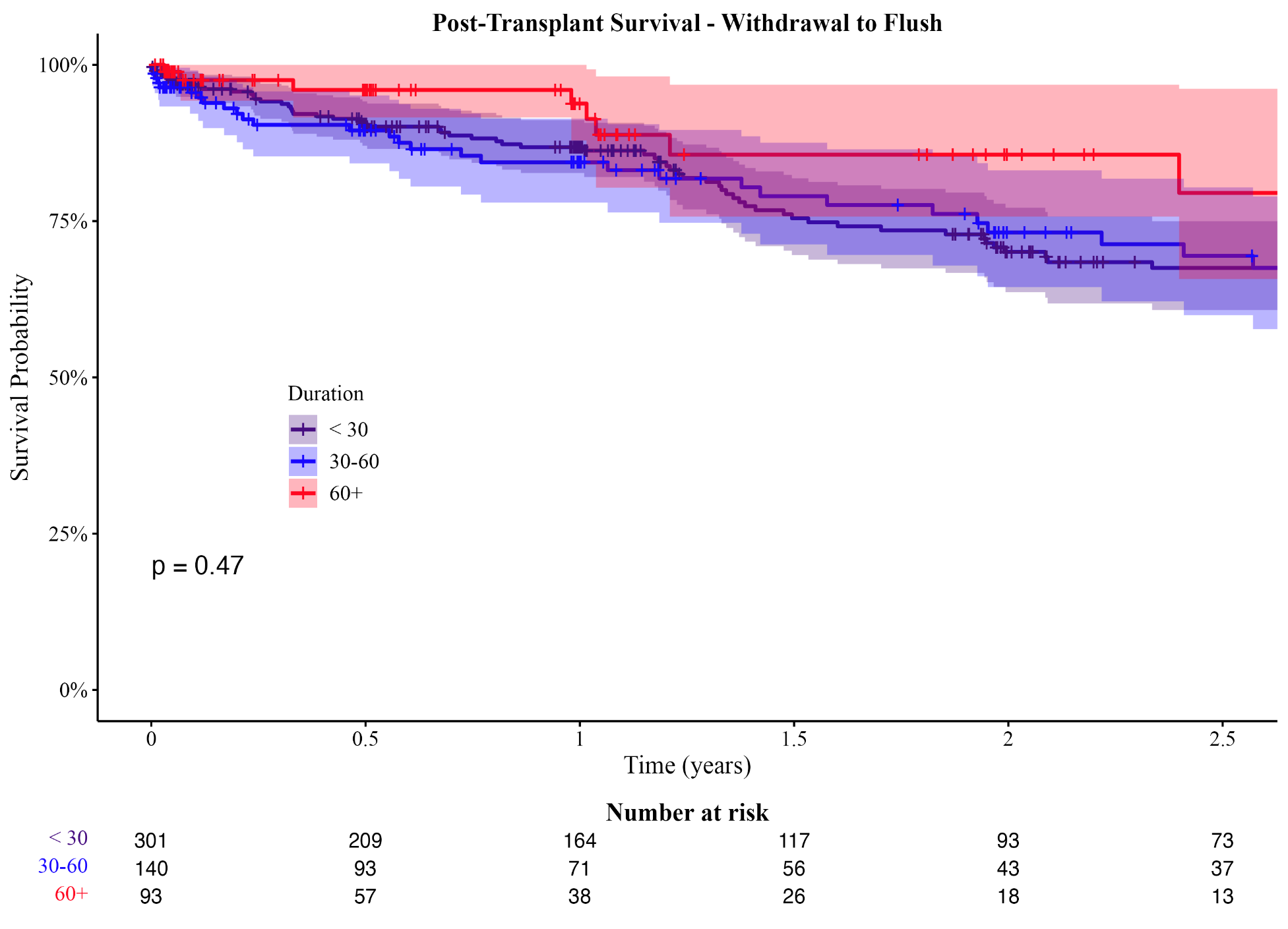
Kaplan-Meier survival curves for 54 months post-transplantation for each withdrawal-to-flush time interval evaluated.

**
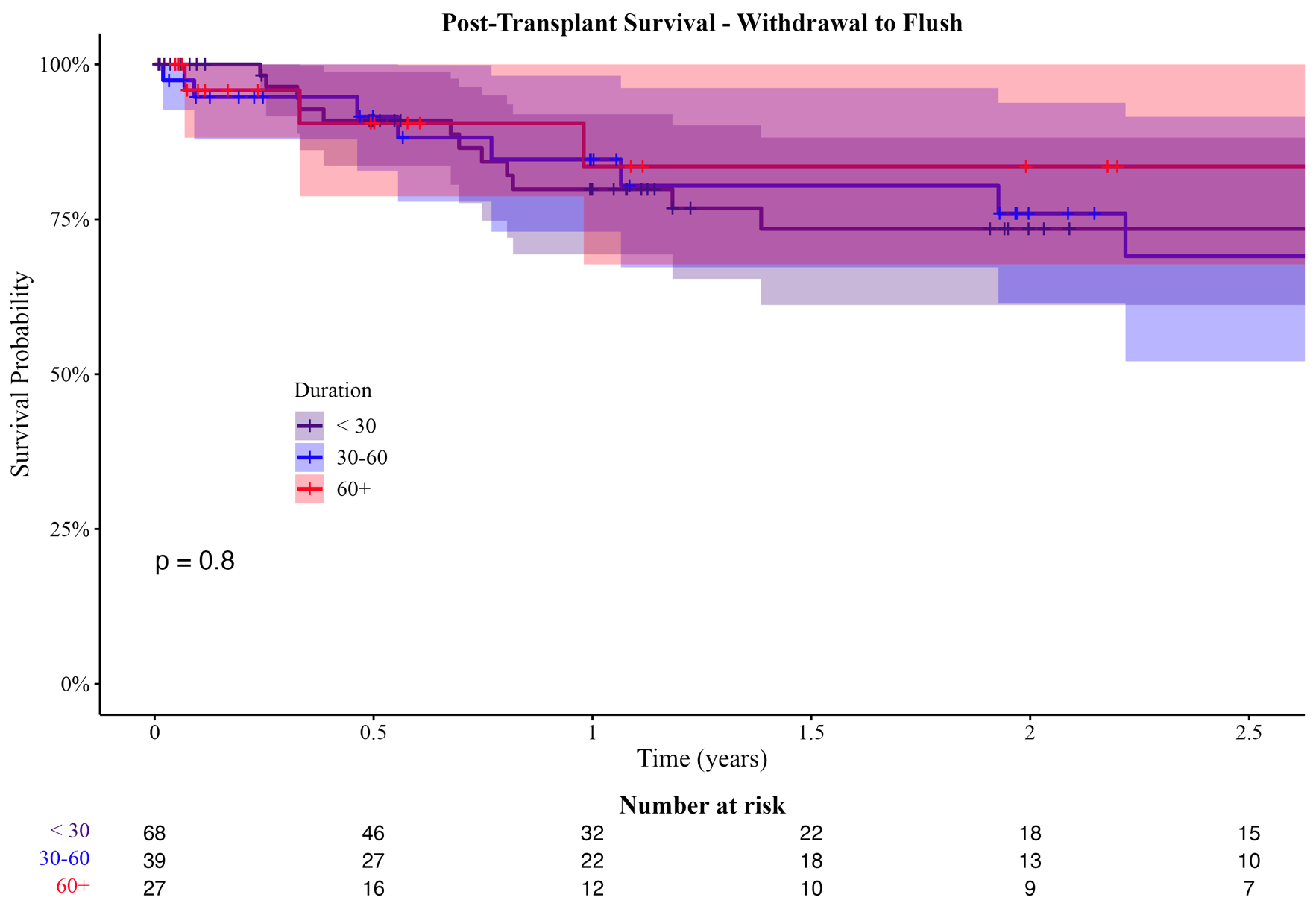
**Kaplan-Meier survival curves for 54 months post-transplantation for each withdrawal-to-flush time interval in EVLP cases evaluated.
